# NAA10 mutation causing a novel intellectual disability syndrome with Long QT due to N-terminal acetyltransferase impairment

**DOI:** 10.1038/srep16022

**Published:** 2015-11-02

**Authors:** Jillian P. Casey, Svein I. Støve, Catherine McGorrian, Joseph Galvin, Marina Blenski, Aimee Dunne, Sean Ennis, Francesca Brett, Mary D. King, Thomas Arnesen, Sally Ann Lynch

**Affiliations:** 1 Clinical Genetics, Temple Street Children's University Hospital, Temple Street, Dublin 1, Ireland; 2 UCD Academic Centre on Rare Diseases, School of Medicine and Medical Sciences, University College Dublin, Dublin 4, Ireland; 3 Department of Molecular Biology, University of Bergen, Norway; 4 Department of Cardiology, Mater Misericordiae University Hospital, Eccles Street, Dublin 7, Ireland; 5 School of Medicine and Medical Sciences, University College Dublin, Dublin 4, Ireland; 6 Department of Neuropathology, Beaumont Hospital, Dublin 9, Ireland; 7 Department of Paediatric Neurology & Clinical Neurophysiology, Temple Street Children’s University Hospital, Dublin 1, Ireland; 8 Department of Surgery, Haukeland University Hospital, Norway; 9 Our Lady’s Children’s Hospital, Crumlin, Dublin 12, Ireland

## Abstract

We report two brothers from a non-consanguineous Irish family presenting with a novel syndrome characterised by intellectual disability, facial dysmorphism, scoliosis and long QT. Their mother has a milder phenotype including long QT. X-linked inheritance was suspected. Whole exome sequencing identified a novel missense variant (c.128 A > C; p.Tyr43Ser) in *NAA10* (X chromosome) as the cause of the family’s disorder. Sanger sequencing confirmed that the mutation arose *de novo* in the carrier mother. *NAA10* encodes the catalytic subunit of the major human N-terminal acetylation complex NatA. *In vitro* assays for the p.Tyr43Ser mutant enzyme showed a significant decrease in catalytic activity and reduced stability compared to wild-type Naa10 protein. *NAA10* has previously been associated with Ogden syndrome, Lenz microphthalmia syndrome and non-syndromic developmental delay. Our findings expand the clinical spectrum of *NAA10* and suggest that the proposed correlation between mutant Naa10 enzyme activity and phenotype severity is more complex than anticipated; the p.Tyr43Ser mutant enzyme has less catalytic activity than the p.Ser37Pro mutant associated with lethal Ogden syndrome but results in a milder phenotype. Importantly, we highlight the need for cardiac assessment in males and females with *NAA10* variants as both patients and carriers can have long QT.

N-terminal (Nt-) acetylation is one of the most common protein modifications in eukaryotes^1^. While its existence has been known for many years, the exact biological role of Nt-acetylation is still unclear. Early evidence suggested that Nt-acetylation may confer protection against protein degradation^2^. More recently, Nt-acetylation has been shown to act as a degradation signal to regulate protein complex stoichiometries^3^,^4^, protein complex formation^5^, subcellular targeting^6^,^7^,^8^ and protein folding^9^. It is likely that the physiological role of Nt-acetylation differs depending on the protein substrate. In humans, six NATs have been identified (NatA-NatF)^10^. NatA is the major NAT complex and modifies approximately 40% of all human proteins^1^. NatA comprises a catalytic subunit encoded by *NAA10* (*ARD1*) and an auxiliary subunit encoded by *NAA15 (NAT1/NATH)*^11^,^12^.

The first reported NAT gene linked to human disease was *NAA10*. In 2011, Rope and colleagues showed that missense mutations in exon 2 of *NAA10* caused Ogden syndrome^13^. Ogden syndrome is a rare perinatal lethal disorder characterised by global developmental delay, craniofacial abnormalities, hypotonia, cardiac arrhythmia and an aged appearance with lax skin^13^. Three years later, Esmailpour and colleagues reported a splice mutation in intron 7 of *NAA10* as the cause of Lenz microphthalmia syndrome (LMS) which includes eye malformations, mild to severe developmental delay and defects in the skeletal and genitourinary systems^14^. That same year, *de novo* mutations in exons 5 and 6 were shown to cause severe non-syndromic developmental delay in an unrelated male and female child^15^. This was the first time that a female with an *NAA10* mutation was shown to have a fully penetrant and severe phenotype. The overlapping but diverse phenotypes associated with *NAA10* were proposed to be allelic disorders with a correlation between the level of remaining Naa10 enzymatic activity and phenotype severity^14^.

We report on two affected brothers from a non-consanguineous Irish family presenting with an intellectual disability syndrome with facial dysmorphism, hypotonia, scoliosis and long QT (Fig. 1 and Table 1). Their mother is also mildly affected suggesting the possibility of X-linked inheritance. More than 100 genes on the X chromosome have been associated with disorders that include X-linked intellectual disability (XLID) as part of the clinical presentation^16^. After negative single gene testing, whole exome sequencing was undertaken to try and establish the genetic cause of the syndrome in this family. We identified a novel variant in *NAA10* (X chromosome) as the cause of this previously unreported syndrome, expanding the clinical spectrum associated with *NAA10* mutation. Functional studies showed reduced acetylation activity of the mutant Naa10 enzyme implicating altered Nt-acetylation as one of the mechanisms contributing to the pathogenesis of syndromic intellectual disability.

## Results

### Clinical report: Patient III:1

**Figure 1:**
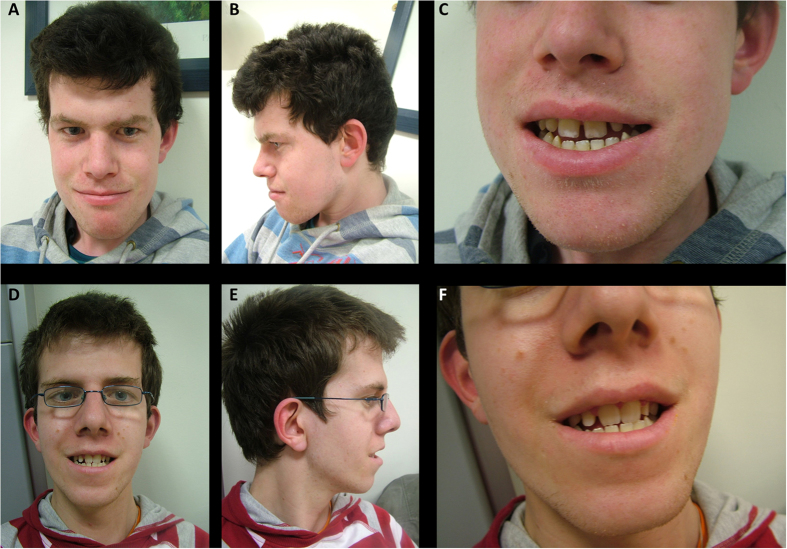
Patient photographs. (**A–F**) Photograph of patient III:1 and III:2 respectively showing (**A,D**) coarse features, depressed midface, hypertelorism, upward tufting or horizontal eyebrows, low set ears, coarse hair, (**B,E**) profile, and (**C,F**) broad teeth.

Patient III:1 was born at term. Perinatal history was unremarkable. He had jaundice and congenital pneumonia in the newborn period and was found to have an innocent heart murmur. Patient III:1 was first noted to have problems in the first year of life when he showed poor weight gain and delay in sitting and standing. Failure to thrive persisted and he continued to grow less than the 3^rd^ centile for height, weight and head circumference. Subsequent investigations identified bilateral acetabular dysplasia which was treated with splints. He took his first step at 23 months and walked at 27 months. Speech was also significantly delayed; he first started using long sentences at 3.5 years. At age 4 years, he showed deterioration in his gait with marked waddling and a persisting tendency to favour his right lower limb. He also had valgus deformity of both heels. Physical examination at 5 years of age identified prominent dysmorphic and coarse features (Fig. 1A–C and Table 1).

Patient III:1 was extensively investigated including evaluation for amino and organic acidopathy, mucopolysarcoidosis, lysosomal storage diseases and liver dysfunction, all of which were negative. Karyotyping of the blood and fibroblasts showed a normal 46 XY karyotype. Cranial nerve examination and fundoscopy were normal and there was no evidence of appendicular ataxia. Computed tomography (CT) showed mild dilation of the lateral ventricles including the temporal horns. The sylvian fissures were a little prominent. There was no focal abnormality or evidence of a white matter disorder. Radiosensitivity testing for ataxia telangiectasia was normal, as was chest X-ray and abdominal ultrasound. He had a normal cardiology exam aged 4 years but electrocardiogram (ECG) was not performed.

At 7 years of age, patient III:1 had three episodes of collapse and loss of consciousness lasting less than 5 minutes. Electroencephalogram (EEG) was abnormal showing mid-line spikes with spread to the right parietal region, consistent with an epileptic focus in that location. The overall background of the EEG was diffusely slow and disorganised, consistent with an encephalopathy.

**Figure 2:**
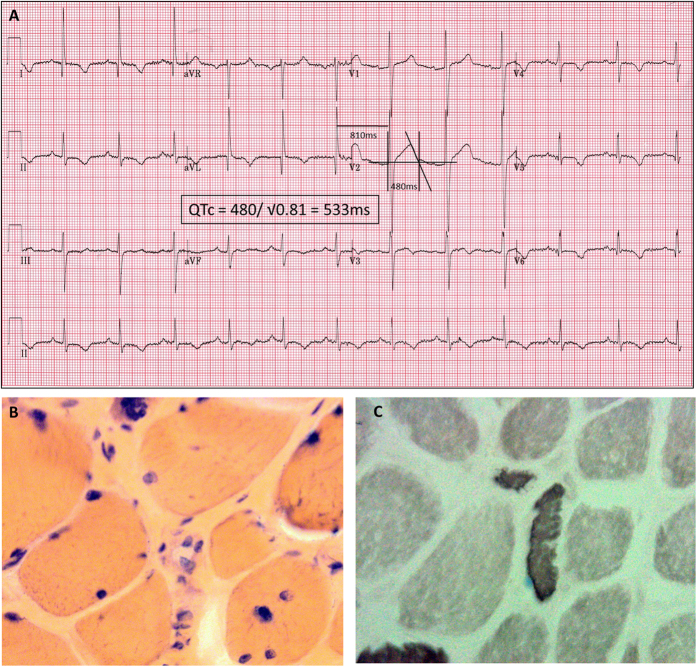
ECG and muscle biopsy. (**A**) Routine ECG on Patient III:1 three years after his presentation with an out-of-hospital cardiac arrest. Left ventricular hypertrophy by voltage criteria with lateral T wave changes are noted, as well as broad-based QT prolongation with a QTc of 533ms (using Bazett’s formula for correction). (**B**) Haematoxylin and eosin (x 40) staining of muscle biopsy from patient III:2 showed variation in fibre size with atrophic fibres. (**C**) Muscle biopsy from patient III:2 showed random atrophic fibers with scattered perivascular lymphocytes. Immunohistochemistry showed that the atrophic fibers were type II with type I fiber predominance.

Patient III:1 came to the attention of the adult cardiology services aged 22 years, when he suffered an out-of-hospital ventricular fibrillation cardiac arrest, which was promptly resuscitated. After recovery, an abnormal ECG was noted with atrial fibrillation, lateral T-wave inversion and repolarisation delay and a QTc of 515 ms. He reverted to sinus rhythm spontaneously but the QT prolongation persisted. Cardiac magnetic resonance imaging (MRI) showed mild concentric left ventricular hypertrophy up to 15 mm, with normal left ventricular function. Beta blockers were commenced and an implantable cardiac defibrillator (ICD) was sited. The patient received an appropriate ICD discharge age 25 years for an episode of sustained polymorphic ventricular tachycardia. Routine ECG age 25 years showed persistence of long QT at 533 ms (Fig. 2A).

### Clinical report: Patient III:2

Patient III:2 was born by Caesarean section for breech at 35 weeks gestation. Similar to his older brother, he had pneumonia, jaundice and failure to thrive in the neonatal period. His facial dysmorphism was identical to that of his older brother but he had additional ophthalmic anomalies (Fig. 1D–F and Table 1). At 8 months of age he presented with acetabular dysplasia and was delayed in sitting, standing and walking. He walked at 27 months and first used meaningful words at 2 years of age. Initially, his gait was normal but by 3.5 years he started to show the same waddling gait as his brother. He was also later found to have lumbar scoliosis, pelvic obliquity, a left-sided inguinal hernia and significantly reduced pulmonary function (likely secondary to muscle weakness and scoliosis). Subsequent history included poor feeding and difficulties with swallowing, recurrent diarrhoea and severe weight loss. His functioning has deteriorated over time with increasing muscle weakness, poor muscle bulk, respiratory difficulties, decreased exercise tolerance and evidence of mild left hemiparesis.

Neurological examination aged 3 showed normal cranial nerves and fundi but detected proximal weakness of the lower limbs (without spasticity). There was some limitation of abduction of the hips bilaterally which may have been related to a problem with muscle contracture. However, tone appeared normal and deep tendon reflexes were present symmetrically. MRI brain showed mild dilation of the ventricles and Magnetic Resonance Spectroscopy (MRS) brain was normal. Electrophysiology examination at 15 years of age showed a complex abnormality with widespread, but not universal, evidence of a membranopathy suggestive of a true myotonia. Motor and sensory nerve conduction velocities were normal as was serum creatine kinase.

Cardiology examination showed a structurally normal heart. However, an ECG identified a superior axis and an RSR pattern in V1. Left ventricular function was normal. A repeat ECG at 17 years of age showed no abnormalities. At 16 years of age, patient III:2 was admitted to hospital with an acute onset of dyspnoea and respiratory distress associated with fever. At 20 years of age, the patient continues to suffer from dyspnoea and bronchospasm, requiring bronchodilators. He has no history of syncope, however his ECG shows conduction system changes with PR prolongation and lateral T wave flattening, with a QTc of 494 ms. He has had no documented ventricular arrhythmia on Holter. No cardiomyopathic changes were appreciated on transthoracic echo or cardiac MRI. With the finding of QT prolongation, beta blockade was added and an implantable loop recorder was sited to monitor for arrhythmia. Rare short runs of atrial fibrillation have been detected on this recorder, though no ventricular arrhythmias to date.

Muscle biopsy showed a striking predominance of type I fibres with rare atrophic type I and type II fibres (Fig. 2B,C). Small fibres were noted to be randomly scattered throughout the muscle fascicles and there were rare perivascular lymphocytes. Ragged red fibres and nemaline rods were not present. Ultrastructural examination of the muscle biopsy revealed a striking increase in the number of subsarcolemmal mitochondria, large clusters of subsarcolemmal mitochondria and dilatation of the sarcoplasmic tubules. Fibroblast culture showed normal fatty acid oxidation and excluded carnitine palmitoyltransferase II deficiency.

### Clinical report: Mother II:2

Individual II:2 has similar facial features to her sons and a mild learning disability, more evident when compared to unaffected family members (Table 1). At the age of 40, she presented with complaints of chest pain and dyspnoea on exertion. She was found to have dyslipidaemia and was known at that time to have hypertension. Coronary angiography revealed premature coronary artery disease, and she subsequently required a number of coronary angioplasty procedures. ECG abnormalities were noted since age 40, with lateral T wave repolarisation changes. Antiplatelet agents were added to her list of therapies. Aged 42, the patient experienced a sudden collapse with loss of consciousness while still on her usual cardiac medications, including a beta blocker. She was again treated with coronary stenting, and a CT brain showed extensive involutional changes. Subsequently, a run of monomorphic ventricular tachycardia was observed on a Holter monitor, and her beta blocker was changed to bisoprolol. She was diagnosed with Type 2 Diabetes Mellitus aged 44 years. At age 48, her ECGs and treadmill ECGs were repeated after the cardiac events which had occurred in her son, and QT prolongation was appreciated, with QTc intervals observed between 460 and 500 ms. Given that her collapse and her non-sustained ventricular tachycardia were seen in the setting of beta blocker use, an ICD was sited (age 48), with no appropriate ICD discharges to date.

### Whole exome sequencing

Whole exome sequencing was performed for one affected child (III:2) and identified 7 novel or rare hemizygous X chromosome variants that were not present in dbSNP, 1000 Genomes, the NHLBI EVS database or our 60 Irish control exomes (Table S1). Three variants were located within genes previously implicated in developmental delay or intellectual disability; a missense variant (rs150861758) in *SHROOM4* (NM_020717.3; c.1879 C > T; p.(Pro627Ser)), a rare missense variant (rs140976011) in *ZCCHC12* (NM_173798.2; c.722 G > C; p.(Arg241Thr)) and a novel missense variant in *NAA10* (NM_001256120.1; c.128 A > C; p.(Tyr43Ser)). *SHROOM4* has been associated with Stocco Dos Santos syndrome (MIM #300434), a disorder characterised by severe mental retardation, delayed/absent speech, short stature, seizures, hyperactivity and aggressive behaviour^17^. The *SHROOM4* p.(Pro627Ser) is a recurrent variant previously identified in families with X-linked mental retardation (XLMR) through exome or panel sequencing^18^,^19^. It is suspected to be benign as segregation analyses have shown that unaffected family members often carry this variant^18^. *ZCCHC12* has been implicated as an XLMR candidate gene and a neurocognitive functional modifier^20^. *NAA10* encodes an N-terminal acetyltransferase associated with variable phenotypes including Ogden syndrome, LMS and non-syndromic developmental delay^13^,^14^,^15^. Of note, the involvement of all three genes in monogenic X-linked intellectual disability (XLID) has recently been challenged. Piton and colleagues concluded that the role of *ZCCHC12* in XLID is questionable while *SHROOM4* and *NAA10* require further confirmatory studies^21^.

### Validation and segregation analyses

**Figure 3:**
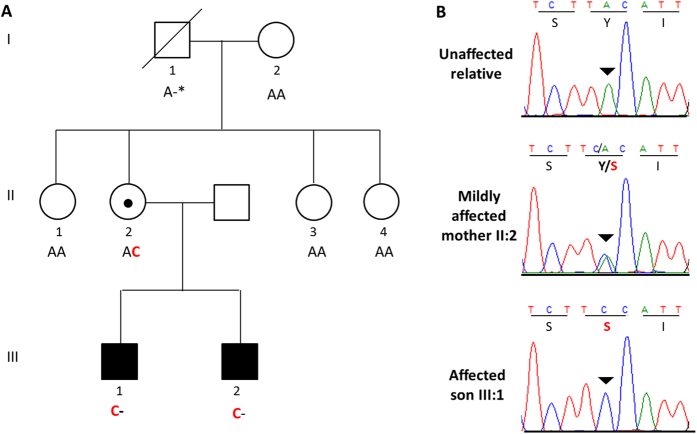
Validation and segregation analysis. (**A**) Sanger sequencing confirmed segregation of the *NAA10* variant with the phenotype and the *de novo* status of the variant in the carrier mother (II:2). *The maternal grandfather’s DNA was not available for analysis. However, sequencing of his unaffected daughters showed that they do not carry the *NAA10* variant on their paternal X chromosome indicating that the grandfather was hemizygous for the reference allele. (**B**) The *NAA10* NM_001256120.1 c.128A > C p.(Tyr43Ser) was validated by Sanger sequence analysis. The inverted triangle indicates the position of the mutated A > C base on the forward strand which results in the substitution of Tyr (Y) with Ser (S) at residue 43.

Sanger sequence analysis of DNA from all available family members excluded *SHROOM4* and *ZCCHC12* as both variants were present in healthy family members (data not shown). Only the novel variant in *NAA10* segregated with the phenotype in a manner consistent with X-linked recessive inheritance; both of the affected males are hemizygous while their mildly affected mother is a heterozygous carrier (Fig. 3). X-inactivation studies in the carrier mother showed a normal non-skewed (random) inactivation pattern in blood. To determine whether the *NAA10* c.128 A > C variant was *de novo* or inherited in the mother (II:2), we sought to sequence the boy’s maternal grandparents (I:1 and I:2). Sanger sequencing confirmed that the maternal grandmother (I:2) did not carry the *NAA10* variant. However, DNA was unavailable from the maternal grandfather (I:1) who is deceased. Therefore, to determine if the variant was *de novo* or paternally inherited in the carrier mother (II:2), we sequenced her three sisters (II:1, II:3 and II:4) as they all inherited the same X chromosome from their deceased father. The sisters did not carry the *NAA10* variant indicating that the variant has occurred *de novo* on the X chromosome of the carrier mother (II:2).

### Naa10 Tyr43Ser mutation modelling

**Figure 4:**
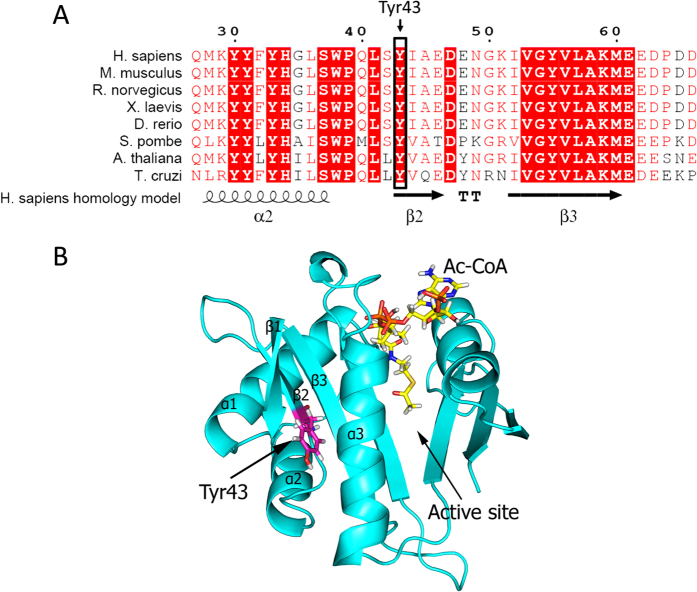
Naa10 homology model and sequence alignment. (**A**) Amino acid sequence alignment of Naa10 orthologues showing that Tyr43 is highly conserved through evolution. Sequences were gathered from the NCBI gene database and the sequence alignment was performed using clustalX. (**B**) Naa10 homology model showing Tyr43 in pink, and Ac-CoA in yellow. Tyr43 is located in the beginning of β2 in the core of the globular domain of Naa10. The side chain of Tyr43 is tightly packed between β1, β3 and α3, with the hydroxyl group facing out towards the surface of the protein.

Naa10 is the catalytic subunit of the NatA complex and is responsible for Nt-acetylation of approximately 40% of the human proteome. Previously described *NAA10* variants identified in patients with non-syndromic developmental delay or Ogden syndrome were shown to have a reduced catalytic activity, while the mutation causing LMS resulted in a loss of Naa10 expression. Amino acid sequence alignment of Naa10 orthologues reveal that Tyr43 is highly conserved through evolution (Fig. 4A), suggesting that Tyr43 is important for Naa10 function. Previously we made a human Naa10 homology model based on the coordinates of *S. pombe* NatA^22^,^23^ which can be used to study the localization of any of the 150 first amino acids of hNaa10. As can be seen in Fig. 4B, Tyr43 is located in the beginning of β2 in the core of the globular domain of Naa10. The side chain of Tyr43 is tightly packed between β1, β3 and α3, with the hydroxyl group facing out towards the surface of the protein. The Naa10 homology model thus suggests that Tyr43 is a structurally important residue for hNaa10, and that a p.(Tyr43Ser) mutation could cause structural changes in Naa10 that either affect Naa10 enzymatic activity or stability.

### *In vitro* analysis of Naa10 Tyr43Ser catalytic activity

**Figure 5:**
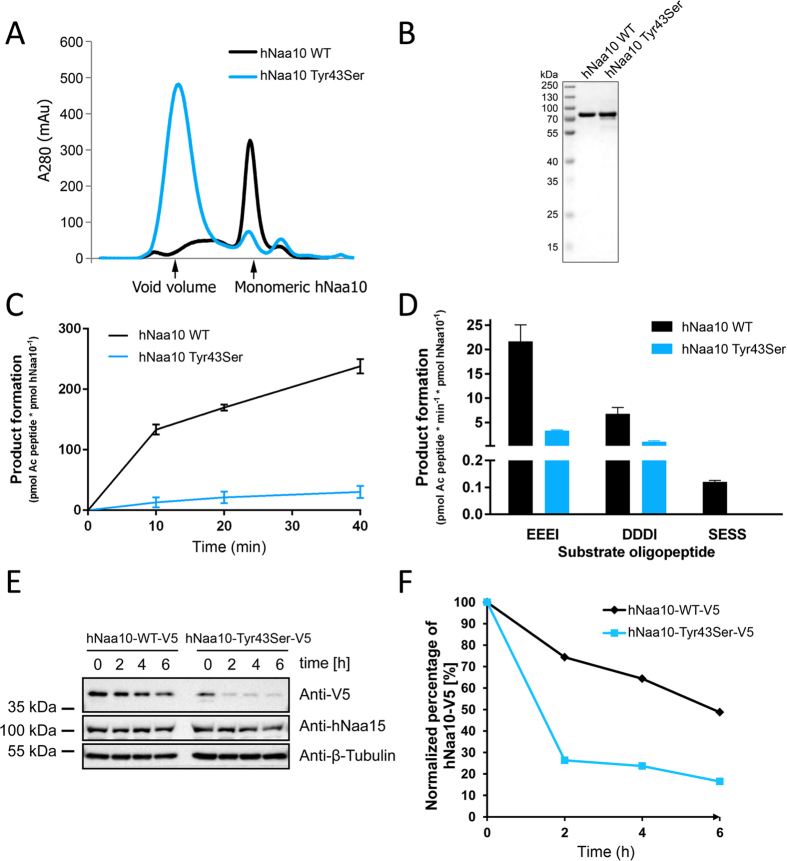
Functional analysis of the hNaa10 Tyr43Ser variant. **(A**) Elution profile from a Superdex200 size exclusion chromatography column. Proteins were expressed in *E. coli* BL21 cells and purified by affinity chromatography and size exclusion chromatography. (**B**) Fractions of monomeric Naa10 WT and Tyr43Ser were collected and the protein concentrations adjusted to equal protein concentrations. In order to assess the purity of the two purified enzymes, samples was analysed by SDS-PAGE and Coomassie. (**C**) Time-dependent NAT assay. Naa10 WT or Tyr43Ser was incubated with 300 μM Ac-CoA and 300 μM oligopeptides. The reaction was stopped at different time points by the addition of quenching buffer (3.2 M guanidinium-HCl, 100 mM sodium phosphate dibasic pH 6.8), and product formation was measured indirectly by the DTNB assay. (**D**) Naa10 NAT activity towards the Naa10 substrates EEEI and DDDI and the NatA substrate SESS. Naa10 Tyr43Ser is clearly catalytically impaired. (**E**) In order to study the stability of hNaa10 WT and hNaa10 Tyr43Ser *in cellulo*, hNaa10-WT-V5 and hNaa10-Tyr43Ser-V5 were expressed in HeLa cells. After two days of growth, cells were treated with 50 μg/ml cycloheximide and the amount of V5-tagged Naa10 present in the sample was measured in the following 6 hours. (**F**) Stability curves illustrating the levels of Naa10-V5 present in cells 0–6 hours following cycloheximide treatment. The stability assay shown is representative of six independent experiments.

In order to directly assess the catalytic activity of the Naa10 Tyr43Ser variant, we mutated plasmids coding for a His/MBP-hNaa10 fusion protein, expressed proteins in *E. coli* BL21 cells and purified hNaa10 WT and hNaa10 Tyr43Ser by affinity chromatography and size exclusion chromatography. As can be seen in Fig. 5A, the largest bulk of Naa10 Tyr43Ser molecules elute in the void volume of the size exclusion chromatography column. This indicates that the protein aggregates in units larger than 200 kDa, most likely due to an alteration of the protein structure, or a reduced protein stability. Despite the large number of molecules eluting in the void volume, a smaller amount of Naa10 Tyr43Ser molecules also eluted as a monomer. Fractions of monomeric Naa10 Tyr43Ser and Naa10 WT were collected and used for enzymatic experiments. In order to obtain similar enzyme concentrations, fractions of Naa10 WT were diluted to the same protein concentration as Naa10 Tyr43Ser, and the purity of the purified enzymes was analyzed by SDS-PAGE and Coomassie (Fig. 5B). Purified enzymes were mixed with Ac-CoA and synthetic oligopeptides representing known Naa10 and NatA substrates and product formation was measured in the linear phase of the reaction. *In vitro* enzymatic assays revealed a drastic reduction of catalytic activity for the Naa10 Tyr43Ser variant. As can be seen in Fig. 5C,D, the Naa10 Tyr43Ser variant is clearly catalytically impaired *in vitro,* with approximately an 85% reduction in catalytic activity for both the Naa10 substrates EEEI and DDDI.

### Analysis of Naa10 Tyr43Ser stability

In order to further assess the stability of the Naa10 Tyr43Ser variant we expressed V5-tagged hNaa10 WT and hNaa10 Tyr43Ser in HeLa cells and performed cycloheximide-chase experiments. As can be seen from Fig. 5E,F, the hNaa10 Tyr43Ser variant is clearly less stable compared to the hNaa10 WT. 2 hours after cycloheximide treatment, the average amount of Naa10 WT molecules present was 67.9 ± 18% relative to the amount of Naa10 molecules before treatment, in comparison to 17.8 ± 8.4% for the hNaa10 Tyr43Ser variant. Altogether our functional analyses reveal a significant functional impairment of the Naa10 Tyr43Ser variant both *in vitro* and *in cellulo*.

## Discussion

We report a novel X-linked recessive intellectual disability syndrome affecting two brothers in a non-consanguineous Irish family. The disorder is characterised by intellectual disability, dysmorphic features, hypotonia, scoliosis and long QT. Exome sequencing identified a missense variant in *NAA10* (c.128 A > C; p.(Tyr43Ser)) as the cause of this syndrome. The affected brothers are hemizygous and inherited the variant from their carrier mother (II:2), who is also mildly affected. Sanger sequencing confirmed that the *NAA10* variant occurred *de novo* in the carrier mother who had normal X-inactivation in blood.

One interesting observation in our study is that the two brothers have remarkable phenotypic differences despite having the same mutation; one boy is more intellectually impaired while the other is more physically disabled. Why ? Intra-familial variability amongst males with X-linked recessive mutations is not common but has previously been reported. Laperuta and colleagues reported marked intra-familial clinical variability in five male relatives with X-linked mental retardation due to *ARX* duplication^24^. Lynch and colleagues described two brothers with a mutation in the X-linked intellectual disability gene *UPF3B* who have variable degrees of developmental delay and notable differences in their spectrum of clinical features^25^. In relation to *NAA10*, there were significant differences in disease severity and progression amongst the nine males with LMS^14^. A number of factors can influence the phenotype and disease penetrance within a family, including gender, age of onset, modifier genes, chance/environmental effects and variations in the ‘rescue’ levels of nonsense-mediated decay (NMD). Gender and age of onset are not relevant in our case as both affected individuals are male and onset was from birth. It is also unlikely that the mutant protein in these patients undergoes NMD as the missense mutation affects protein structure and activity but does not produce a truncated protein. The exact reason for the variability between these two brothers is unknown but may relate to modifier effects of variants in another gene and non-genetic factors.

**Figure 6:**
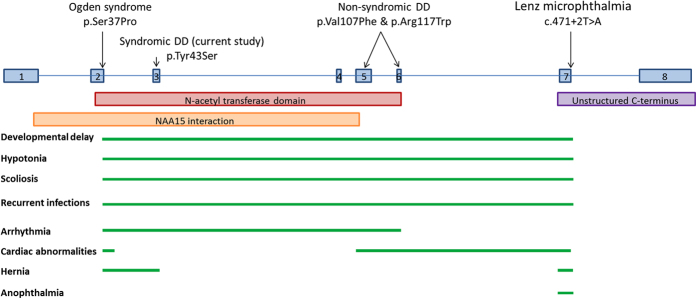
Schematic outlining the genotype-phenotype correlation. The location of each reported *NAA10* variant associated with a specific syndrome is indicated above the schematic of the *NAA10* gene (8 exons). The annotated domains and interaction sites are noted below the gene. The presence of a particular clinical feature is indicated by a green bar.

Mutations in *NAA10* have previously been associated with Ogden syndrome, LMS and non-syndromic developmental delay which are believed to be allelic disorders^13^,^14^,^15^. We now expand the *NAA10* clinical spectrum to include a syndromic developmental delay disorder with long QT. Examination of the clinical features associated with all four *NAA10*-related disorders suggests a possible genotype-phenotype correlation (Fig. 6 and Table 2). To date, all patients with *NAA10* mutations show four common clinical features regardless of their syndrome; developmental delay (ranging from mild to severe), hypotonia, scoliosis and recurrent infections. So far, only patients with mutations in exons 2-6 (all those except patients with LMS) have experienced cardiac arrhythmias. Of note, all of the arrhythmia-related mutations are located within the N-acetyl transferase domain of *NAA10*. Conversely, the LMS mutation which does not cause an arrhythmia is located outside of the N-acetyl transferase domain. This observation hints at a possible interaction between the *NAA10* N-acetyl transferase domain and a known or novel arrhythmia gene. Alternatively, the reduced N-acetyl transferase activity of the mutant Naa10 enzymes may have a downstream effect on the regulation of an arrhythmia gene or protein. Further studies looking at differential gene expression may help to decipher the basis for the cardiac arrhythmias in these patients.

As Naa10 is thought to acetylate over 8,000 different proteins, each disease mutation may affect the acetylation of different sets of substrates, resulting in different phenotypic features. Furthermore, one or more of the proposed lysine acetyltransferase (KAT) mediated activities or acetyltransferase independent functions of Naa10 may be affected^26^. Our functional studies revealed both a reduced stability and catalytic activity of the Naa10 Tyr43Ser variant. Naa10 Tyr43Ser had an approximately 85% reduction in catalytic activity compared to hNaa10 WT. In comparison, a 20-75% (dependent on the substrate oligopeptide tested) reduction in catalytic activity was measured for the hNaa10 Ser37Pro variant causing OS, and an approximately 15% reduction in catalytic activity measured for the hNaa10 R116W variant causing NSDD in a 6 year old boy. Given the clinical features in the family reported in this study, it is surprising that Naa10 Tyr43Ser results in a more severe impairment in catalytic activity compared to the Ogden syndrome mutant Naa10 Ser37Pro which is lethal. Furthermore, Naa10 Tyr43Ser is unstable as compared to Naa10 wildtype, a feature not observed for Naa10 Ser37Pro^23^,^27^. It was previously suggested that the remaining catalytic activity of Naa10 variants could correlate with the severity of the phenotypes observed in each patient^15^. However, the present study shows that the relation between Naa10 mutations and disease phenotypes is more complex and that the *in vitro* catalytic activity in itself is not always sufficient in explaining phenotypes observed in patients.

There are a number of potential reasons that could account for these apparently contradicting observations. Firstly, the actual *in vivo* activity of Naa10 Tyr43Ser could be higher than that of Naa10 Ser37Pro (due to stabilizing partners or otherwise) and the *in vivo* Nt-acetylome is only modestly affected in Naa10 Tyr43Ser cells. Secondly, Ogden syndrome may be caused by Nt-acetylation defects plus additional not yet elucidated non-NAT mediated effects. In contrast, Naa10 Tyr43Ser cells may only be defective in their Nt-acetylation. Thirdly, genetic background and proteome profile variation may modulate the Nt-acetylation capacity of the individual and cause differences in the impact of Nt-acetylation defects. For example, individual differences in the expression of Naa11 (ARD2), a functional Naa10 paralogue^28^, could have a significant impact on NatA-mediated Nt-acetylation. Lastly, the *NAA10* gene has 16 transcripts (Ensembl) of various lengths and starting positions. Therefore, depending on the location of a mutation, it may impact all or only a subset of *NAA10* transcripts, which could influence the severity of the resulting phenotype.

The *NAA10* variants causing non-syndromic developmental delay are *de novo* while the variants causing Ogden syndrome, LMS and syndromic development delay (current study) were inherited from carrier mothers. With Ogden syndrome, the carrier mothers have significantly skewed patterns of X-inactivation (90-100%) biased against the X chromosome carrying the mutation, thereby protecting them from phenotypic expressivity^23^. With LMS, the heterozygous female carriers have mild manifestations such as abnormal ears and syndactyly which are milder versions of features present in the affected males^14^. While X-inactivation was not analysed in the carrier females, it was suggested that these mild features could be due to reduced expressivity or X-chromosome skewing with preferential inactivation of the wild-type allele^14^. In the current study, the carrier mother has mild manifestations of the disorder in her sons including long QT and mild intellectual disability. A normal non-skewed X-inactivation pattern was observed in blood. Therefore, the mild features in the mother may be due to tissue-specific X-chromosome skewing or a dominant-negative effect of the mutant protein.

In summary, we report a novel missense variant in *NAA10* as the cause of a new developmental delay syndrome which includes long QT. Our findings suggest that it is important to consider cardiac screening in both male and female patients who have pathogenic *NAA10* mutations as even female carriers can have long QT. As is the case in our study, female carriers may have a predominantly cardiac phenotype and it may not be obvious that their cardiac problems are part of a syndrome. Therefore, consideration should be given to the addition of the *NAA10* gene to Next-Generation Sequencing-based cardiac panels. To date, *NAA10* has been implicated in a variety of overlapping yet distinct disorders – all of which are likely caused by impaired Nt-acetylation of NatA targets. It is possible that each disease mutation has different effects on the acetylation of NatA’s 8,000 protein targets, thereby accounting for the diverse range of clinical features associated with this gene. Further investigation into the spectrum of downstream acetylation defects in each syndrome may help to understand how variation in a single gene can give rise to such complex phenotypes.

## Materials and Methods

### Consent and ethics

Written informed consent, including consent to publish patient photographs, was obtained from the patients. The study protocol was approved by the ethics committee of Temple Street Children’s University Hospital (Dublin, Ireland). All experiments were performed in accordance with the approved guidelines and regulations.

### Whole exome sequencing

Genomic DNA was isolated from blood lymphocytes according to standard procedures. DNA from one affected child (III:2) was selected for whole exome sequencing (Eurofins Genomics, Germany). The exonic DNA was enriched with the SureSelectv5 50 Mb Human All Exon Kit (Agilent Technologies, Santa Clara), and sequenced on an Illumina HiSeq at Eurofins Genomics (Ebersberg, Germany). The 100 bp paired-end reads were aligned to the hg19 human reference genome with the Burrows-Wheeler Alignment tool 0.5.7^29^. Reads of inadequate sequence quality and potential PCR duplicates were discarded. The quality scores for the aligned reads were recalibrated using GATK^30^. Regions containing clusters of SNPs were identified and the reads in these regions were realigned using GATK. Variants and indels were identified using SAMtools and annotated using ANNOVAR^31^. Assuming an X-linked recessive model, we prioritised variants that were (i) absent or present with a frequency < 1% in dbSNP142, 1000 Genomes and the NHLBI Exome Variant Server database (http://evs.gs.washington.edu/EVS/), (ii) coding (nonsense, missense, splice site or indel), (iii) on the X chromosome (X-specific), (iv) hemizygous, (v) absent in our 60 control exomes, (vi) previously implicated in developmental delay/intellectual disability and (vii) segregated with the phenotype (Table S2).

### Validation and segregation analysis

Candidate variants were validated and segregation tested by bi-directional Sanger sequencing of DNA from all available family members (Table S3). As DNA was unavailable from the deceased father (I:1) of patient II:2, we confirmed the *de novo* status of the candidate *NAA10* variant by testing the sisters of patient II:2, all of whom would have inherited the same paternal X chromosome as the patient. If the sisters carried the variant it would indicate paternal inheritance. Conversely, absence of the variant from the X chromosome of the sisters would confirm that the maternal grandfather (I:1) did not carry the candidate variant on his X chromosome and, hence, it is *de novo* in patient II:2. Patient II:2 and an unaffected sibling were genotyped for 964,193 Single Nucleotide Polymorphisms (SNPs) on the Illumina OmniExpress Exome array according to standard protocols (AROS Applied biotechnology, Aarhus, Denmark). Family structure was confirmed using identity-by-state calculations in PLINK^32^. The identified *de novo* variant in *NAA10* was submitted to the LOVD gene variant database at http://www.lovd.nl/NAA10.

### Plasmid construction and protein purification

Plasmids encoding His/MBP-Naa10 p.Tyr43Ser, and V5-Naa10 p.Tyr43Ser were created by site-directed mutagenesis (QuikChange® Multi Site-Directed Mutagenesis kit) according to the manufacturer’s protocol. A pETM41 vector (from G. Stier, EMBL, Heidelberg, Germany) encoding Naa10 WT with Maltose Binding Protein/His-fusion as a fusion tag^33^, and a pcDNA3.1 mammalian expression vector (Life technologies) encoding Naa10 WT with a V5 fusion tag^12^ were used as a template for the site-directed mutagenesis. Primers used for the mutagenesis were hNAA10 A128C f: CCTGGCCCCAGCTCTCTTCCATTGCTGAGGACGAGAATGGG and hNAA10 A128C r: CCCATTCTCGTCCTCAGCAATGGAAGAGAGCTGGGGCCAGG. Mutations were verified by Sanger sequencing, and plasmids were transformed into *E. coli* BL21 Star (DE3) by heat shock transformation. *E. coli* BL21 cells were grown in 200 mL cell cultures at 37 °C to an OD600nm of 0.6, and then cooled down to 18 °C before starting protein expression by the addition of IPTG to a total concentration of 500 μM. After 14 hours of incubation, cell cultures were harvested and the pellet stored at −80 °C. Pellets were dissolved in lysis buffer (50 mM Tris-HCl (pH 7.4), 1 M NaCl, 1 mM DTT, and one tablet of EDTA-free protease inhibitor per 50 mL) and cells lysed by 6 × 30 seconds of sonication on ice. Recombinant MBP-hNaa10 was purified by Immobilized Affinity Chromatography (1ml HisTrap HP, GE Healthcare) and Size Exclusion Chromatography (Superdex 200 10/300, GE Healthcare). Buffers used for purification were: IMAC wash buffer (50 mM Tris-HCl (pH 7.4), 300 mM NaCl, 1 mM DTT, 25 mM Imidazole), IMAC elution buffer (50 mM Tris-HCl (pH 7.4), 300 mM NaCl, 1 mM DTT, 300 mM Imidazole) and Size exclusion chromatography buffer (50 mM HEPES (pH 7.5), 300 mM NaCl, 1 mM DTT). Fractions were analyzed by SDS-PAGE and protein concentration was determined by both A280 measurements (Nanodrop1000) and Bradford assay (BioRad).

### *In Vitro* Acetylation Assays

As previously described, a colorimetric acetylation assay^34^,^35^ and a quantitative HPLC assay^36^ were used to measure the catalytic activity of the Naa10 variants. Briefly for the colorimetric assay, the thiol present in the enzymatic product, CoA, cleaves 5,5′-dithiobis-(2-nitrobenzoic acid) (DTNB) and produces 2-nitro-5-thiobenzoate (NTB^–^), which is quantified by monitoring the absorbance at 412 nm. Purified enzymes were mixed with different substrate peptides and Ac-CoA in acetylation buffer (50 mM HEPES pH 7.5, 100 mM NaCl, and 0.2 mM EDTA), and reactions were quenched with quenching buffer (3.2 M guanidinium-HCl, 100 mM sodium phosphate dibasic pH 6.8). As a negative control, reactions without enzyme were incubated at 37 °C, and enzymes were added to the reaction after quenching. To measure CoA production, DTNB (2 mM final, dissolved in 100 mM sodium phosphate dibasic pH 6.8 and 10 mM EDTA) was added to the quenched reaction and the absorbance at 412 nm was measured. Background absorbance was determined in the negative controls, and subtracted from the absorbance determined in each individual reaction. Thiophenolate production was quantified assuming ε = 13.7 × 10^3^ M^–1^ cm^–1^.

In the quantitative HPLC acetylation assay, enzymes were mixed with substrate peptides and Ac-CoA in reaction buffer (50 mM HEPES pH 7.5, 100 mM NaCl and 0.2 mM EDTA), incubated at 37 °C and stopped by the addition of 10% Trifluoroacetic acid (TFA). Product formation was quantified with RP-HPLC as described previously^36^. In the quantitative acetylation assay the following oligopeptides (Biogenes) were used: EEEI ([H]EEEIAAL RWGRPVGRRRRPVRVYP[OH]) representing γ-actin, DDDI ([H]DDDIAAL RWGRPVGRRRRPVRVYP[OH]) representing β-actin and SESS ([H]SESSSKS RWGRPVGRRRRPVRVYP[OH]), representing high-mobility group protein A1. Product formation was measured in the linear phase of the reaction.

### Protein stability analysis of hNaa10-Tyr43Ser-V5 by cycloheximide chase assay

HeLa cells were plated on 6 well plates (Ø 34.8 mm) 16 h before transfection. Cells at 80% confluency were transiently transfected with 2.6 μg hNaa10-Tyr43Ser-V5 and hNaa10-WT-V5, respectively by using X-tremeGENE 9 DNA transfection reagent (Roche). After 24 h medium was replaced. 48 h after transfection 50 μg/ml cycloheximide was added to the cells. For samples of time point 0 h no cycloheximide was added to the cells before harvesting. 2 h, 4 h and 6 h after adding cycloheximide, cells were harvested in 1400 μl cold PBS (pH 7.4) by using a cell scraper. Cells were spun down at 4 °C and 1000 × g for 5 minutes. Supernatant was removed before cells were resuspended in 1 ml of cold PBS. Cells were again spun down under same conditions. After removing of supernatant, cells were lysed in 40 μl of IPH lysis buffer (50 mM Tris-HCl pH 8.0, 150 mM NaCl, 5 mM EDTA, 0.5% NP-40), containing complete EDTA free protease inhibitor (Roche), on ice for 20 minutes. After centrifugation at 4 °C and 17000 x g for 8 minutes, supernatant was transferred into a new reaction tube and Laemmli loading buffer, containing SDS, was added. The V5-tagged hNaa10 protein was detected using a standard Western blotting procedure. Therefore, 10 μl of each sample was resolved on a 12% SDS-PAGE gel, transferred onto a nitrocellulose membrane (Amersham Protran 0.2 NC) and after blocking with 5% non-fat dry-milk (Regilait), incubated with anti-V5-tag antibody (Invitrogen, R960-25), anti-β-Tubulin antibody (Sigma Aldrich, T5293), anti-hNaa10 antibody (anti-hARD1) and anti-hNaa15 antibody (anti-NATH)^12^. The blot was visualized using horseradish peroxidase-conjugated anti-mouse IgG antibody and anti-rabbit IgG antibody (GE Healthcare) respectively and SuperSignal West Pico Chemiluminescent Substrate Kit (Thermo Scientific). Signals were detected by using ChemiDoc^TM^ XRS + system of Bio-RAD. Normalization of the signals was done with ImageLab^TM^ system 5 of Bio-RAD.

## Additional Information

**How to cite this article**: Casey, J. P. *et al.* NAA10 mutation causing a novel intellectual disability syndrome with Long QT due to N-terminal acetyltransferase impairment. *Sci. Rep.* **5**, 16022; doi: 10.1038/srep16022 (2015).

## Supplementary Material

Supplementary Information

## Figures and Tables

**Table 1:** Clinical features in Irish family with X-linked intellectual disability syndrome.

Clinical feature	III:1	III:2	II:2 (mother)
Neonatal jaundice	yes	yes	not known
Growth parameters	height < 0.4^th^ct, weight < 0.4^th^ct, OFHC < 3^rd^ct	height 25^th^ct, weight < 25^th^ct, OFHC 91^st^ct,	height 10^th^ ct
Intellectual disability	moderate	mild	Mild
Facial			
Dysmorphism	coarse features, low anterior headline coming to a peak in the midline, hypertelorism, epicanthic folds, prominent philtrum and forehead with high cheek bones, depressed mid face, snub nose, flat nasal bridge, low set and small crumpled ears, coarse hair	similar dysmorphic features to brother III:1 with mild posterior rotation of ears	similar facial features to her sons; small ears, coarse hair
Grommet insertion and adeno/tonsillectomy	yes (age 3.5 years)	yes (age 2 years)	no
Eye morphology	normal	Normal	Normal
Eye anomalies	not tested	moderate RCS, HA, dense RA	Normal
Hypertelorism	yes	no	No
Eyebrows	pointy, upward tufting	horizontal	Normal
Downslanting palpebral fissures	yes, epicanthic folds	yes	Yes
Skeletal			
Syndactyly	2/3 toe syndactyly	no	no
Scoliosis	mild; lower spine	right lumbar	no
Abnormal teeth	broad front teeth, hyper -calcification 6 lower	broad front teeth	broad front teeth
Small hands/feet	yes; shoe size 38	yes; shoe size 38	yes; size 37.5
Feet abnormalities	VD of both heels	no	no
Hips	marked BAD	BAD, PO	no
Inguinal hernia	yes	yes	no
Cardiac			
Arrhythmias	yes; two MI	yes	yes
Prolonged QT interval	yes	yes	yes
Other cardiac features	innocent HM at birth	innocent HM, VT, superior axis ECG	premature CAD, VT
Neurological			
Hypotonia	yes	yes; progressive muscle weakness, poor muscle bulk, mild left HP	no
Seizures	yes	no	no
MRI	mild dilation of LV, mild CA, prominent SF	mild dilation of LV, mild CA	not tested
Other features	congenital pneumonia, sacral dimple, markedly distended veins over chest and upper abdomen, remarkably prominent vascular pattern over skull	distended veins on chest, upper abdomen and around nipples, long history of perennial rhinitis, recurrent sinusitis with extensive nasal polyps and spasmodic cough	coeliac disease from childhood, type 2 diabetes, Graves disease

Patients III:1 and III:2 are brothers who presented with a developmental delay syndrome and long QT. Their mother (II:2) is also mildly affected. Abbreviations: BAD: bilateral acetabular dysplasia; CA: cerebral atrophy; CAD: coronary artery disease; HA: hyperopic astigmatism; HM: heart murmur; HP: hemiparesis; LV: lateral ventricles; MI: myocardial infarctions; PO: pelvic obliquity; RA: right amblyopia; RCS: right convergent squint; SF: sylvian fissures; VD: valgus deformity; VT: ventricular tachycardia.

**Table 2:** Comparison of clinical features present in *NAA10*-associated disorders.

Clinical feature	OS	LMS	NSDD	Current
Developmental delay	+++	++	+++	+
Delayed motor development	+	+	+	—
Aged appearance	+	—	—	—
Microcephaly	—	—	+	—
Craniofacial abnormalities	+	—	+	—
Dysmorphic features	++	++	+	+++
Fontanels	large	—	delayed closure	—
Microretrognathia	+	—	—	—
Eyes	prominent	small or absent	deep set	normal
Hypertelorism	+*	—	—	+*
Truncal hypotonia	+*	+	+	+
Hypertonia	+	—	+	—
Abnormal ears	large	over-folded upper pinnae	large	low set, crumpled
Flared nares	+	—	—	—
Abnormal palate	narrow	high arched	high arched	—
Pectus abnormalities	excavatum	excavatum	carinatum	—
Clinodactyly	+	+	—	—
Foot and toe abnormalities	+	+	+	+*
Small hands and feet	—	—	+	+
Scoliosis	+++	+	+	++
Renal abnormalities	—	+*	—	—
Cardiac arrhythmia	+	—	+	+
Cardiac malformations	ASD, PPS, CM, DA	+	PAS, ASD	—
Cryptorchidism	+	—	—	—
Hernia	Umb, Ing	diaphragmatic*	—	umbilical
Little subcutaneous fat	+	—	—	—
Eczema	+	—	—	—
Recurrent infections	+	+	+	+
Otitis media	+	+(chronic)	—	+(recurrent)
Seizures	+*	+*	—	+*
Lethality	+	—	—	—
Behavioural issues	n/a	+++	++	—
X chr skewing in females	+++	+	n/a	—

Clinical features reported in all four *NAA10*-associated disorders are highlighted in bold. Features are denoted as present (^+^mild, ^++^moderate, ^+++^severe) or absent (–). Features present in some but not all patients with that particular disorder are denoted by the * symbol. Abbreviations: ASD: atrial septal defect; CM: cardiomegaly; DA: ductus arteriosus; Ing: inguinal; LS: Lenz microphthalmia; NSDD: non-syndromic developmental delay; OS: Ogden syndrome; PAS: pulmonary artery stenosis; PPS: peripheral pulmonary stenosis; Umb: umbilical
