# Supplementary material for: NAA10 mutation causing a novel intellectual disability syndrome with Long QT due to N-terminal acetyltransferase impairment
Source: Sci Rep. 2015 Nov 2;5:16022. doi: 10.1038/srep16022 (PMC4629191; doi:10.1038/srep16022)
Supplement: Supplementary Information [file srep16022-s1.pdf]

## Supplementary Information

# NAA10 mutation causing a novel intellectual disability syndrome with Long QT due to N-terminal acetyltransferase impairment

Jillian P. Casey[1,2*], Svein I. Støve[3], Catherine McGorian[4,5], Joseph Galvin[4], Marina Blenski[3], Aimee Dunne[2], Sean Ennis[2], Francesca Brett[6], Mary D. King[7,2], Thomas Arnesen[3,8], Sally Ann Lynch[1,2,9]

1. Genetics Department, Children's University Hospital, Temple Street, Dublin 1, Ireland.
2. UCD Academic Centre on Rare Diseases, School of Medicine and Medical Sciences, University College Dublin, Dublin 4, Ireland.
3. Department of Molecular Biology, University of Bergen, Norway.
4. Department of Cardiology, Mater Misericordiae University Hospital, Eccles Street, Dublin 7, Ireland.
5. School of Medicine and Medical Sciences, University College Dublin, Dublin 4, Ireland.
6. Department of Neuropathology, Beaumont Hospital, Dublin 9, Ireland.
7. Department of Paediatric Neurology & Clinical Neurophysiology, Temple Street Children's University Hospital, Dublin 1, Ireland.
8. Department of Surgery, Haukeland University Hospital, Norway.
9. Our Lady's Children's Hospital, Crumlin, Dublin 12, Ireland.

**Table S1. Rare or novel X-specific variants identified by whole exome sequencing in patient III:1.**

| **Gene** | **Transcript** | **cDNA alteration** | **Protein alteration** | **dbSNP ID** | **MAF (%)** | **Implicated in DD/ID** | **Variant segregates** |
|---|---|---|---|---|---|---|---|
| *SHROOM2* | NM_001649.2 | c.4274C>T | p.(Ser1425Leu) | rs149373874 | 0.81 | no | - |
| *SHROOM4* | NM_020717.3 | c.1879C>T | p.(Pro627Ser) | rs150861758 | 0.29 | yes | no |
| *IL13RA1* | NM_001560.2 | c.597_599delAAC | p.(Gln199del) | novel | - | no | - |
| *ZCCHC12* | NM_173798.2 | c.722G>C | p.(Arg241Thr) | rs140976011 | 0.06 | yes | no |
| *GPR112* | NM_153834.3 | c.6217C>A | p.(Pro2073Thr) | rs371661996 | 0.01 | no | - |
| *SPANXD* | NM_032417.2 | c.220A>G | p.(Lys74Glu) | rs2983592 | unknown | no | - |
| *MAGEA4* | NM_001011550.1 | c.122C>T | p.(Ser41Phe) | rs41302158 | 0.46 | no | - |
| *NAA10* | NM_001256120.1 | c.128A>C | p.(Tyr43Ser) | novel | - | yes | yes |

Variant prioritisation identified eight hemzygous coding variants on the X chromosome of patient III:1 which were novel or had a minor allele frequency (MAF) less than 1% in control databases and Irish controls. Three of the eight genes have previously been implicated in developmental delay (DD) or intellectual disability (ID). Only one of those three remaining candidates segregated with the phenotype in this family.

**Table S2. Whole exome sequencing variant prioritisation strategy**

| **Criteria** | **Number of Variants** |
|---|---|
| Variants identified | 203,992 |
| + absent or present with a frequency <1% in dbSNP130, NHLBI EVS and 1000G | 27,486 |
| + Nonsense, missense, splice site or indel | 1,260 |
| + X Chromosome | 27 |
| + hemizygous and X-specific | 11 |
| + absent or present with a frequency <1% in 60 Irish control exomes | 7 |
| + previously implicated in developmental delay or intellectual disability | 3 |
| + segregates with the phenotype | 1 |

Rare or novel hemizygous variants in genes on the X chromosome implicated in developmental delay or intellectual disability were prioritised. Of the three candidate variants, only one variant segregated with the phenotype; a novel missense variant (c.128A>C; p.(Tyr43Ser)) in the *NAA10* gene.

**Table S3. Primer sequences for PCR.**

| Gene | Exon number | Forward primer 5’-3’ | Reverse primer 5’-3’ | Size (bp) | Annealing temperature |
|---|---|---|---|---|---|
| *SHROOM4* | Exon 4 | CACAGCAGCCACAAAGGG | TGAAGAGCTTGTCTCTGGGG | 613 | 60° |
| *ZCCHC12* | Exon 4 | CAAACCGGACTCGCTTGCAG | CCTGGGCCTTGTCACTCTCG | 597 | 60° |
| *NAA10* | Exon 3 | ACTCGTCCAGTTTGTGTCCC | ACAGAGGCATCCACCAGAC | 192 | 60° |

PCR primer sequences were designed using the Exon Primer programme available in the UCSC Genome Browser.
